# Supplementary material for: Targeting Vesicular LGALS3BP by an Antibody-Drug Conjugate as Novel Therapeutic Strategy for Neuroblastoma
Source: Cancers (Basel). 2020 Oct 15;12(10):2989. doi: 10.3390/cancers12102989 (PMC7650653; doi:10.3390/cancers12102989)
Supplement: Supplementary file 1 [file cancers-12-02989-s001.zip › cancers-931468-XML-suppl.docx]

Supplemental material

Targeting Vesicular LGALS3BP by an Antibody-Drug Conjugate as Novel Therapeutic Strategy for Neuroblastoma

Emily Capone, Alessia Lamolinara, Fabio Pastorino, Roberta Gentile, Sara Ponziani, Giulia Di Vittorio, Daniela D’Agostino, Sandra Bibbò, Cosmo Rossi, Enza Piccolo, Valentina Iacobelli, Rossano Lattanzio, Valeria Panella, Michele Sallese, Vincenzo De Laurenzi, Francesco Giansanti, Arturo Sala, Manuela Iezzi, Mirco Ponzoni, Rodolfo Ippoliti, Stefano Iacobelli and Gianluca Sala


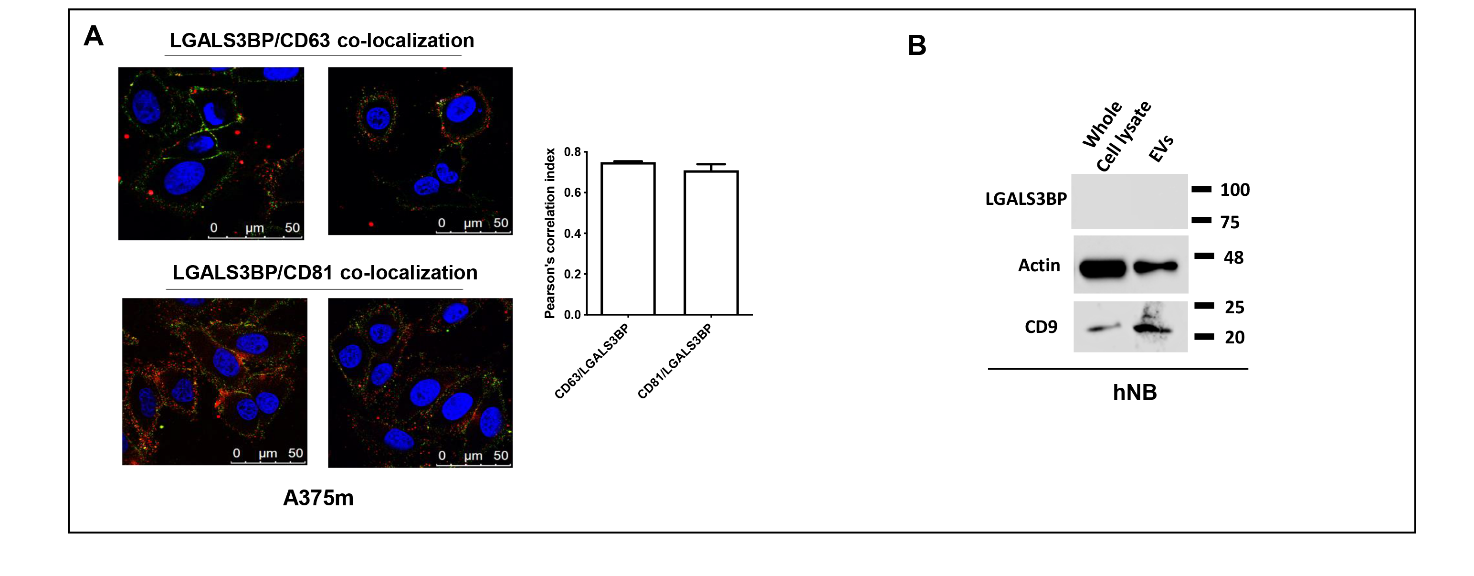


**Figure S1.** LGALS3BP co-localizes with EVs markers (**A**) Confocal imaging showing LGALS3BP (red) co-localization with CD63 and CD81 (green) in A375m melanoma cells. Cells nuclei were stained by DRAQ5 (blue). (**B**) Immunoblot showing exosomal markers Actin and CD9 expression in EVs isolated from hNB cell culture supernatant compared to whole cell lysate.


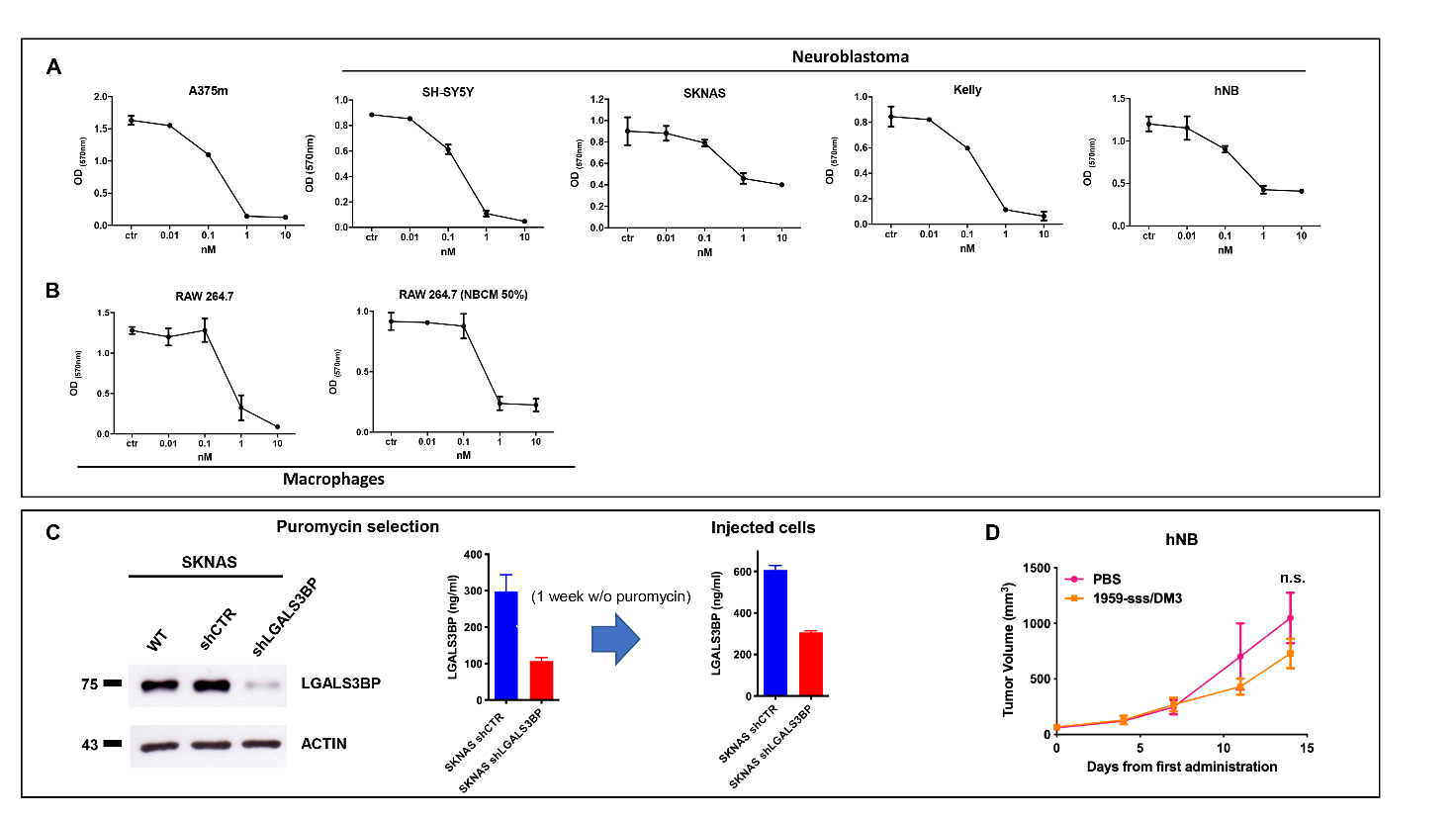


**Figure S2.** SH-DM3 in vitro cell killing activity (**A**) In vitro cell killing activity of free SH-DM3 was evaluated in a panel of neuroblastoma cell lines by MTT assay after 72 h of treatment with the indicated doses. SH-DM3 sensitive A375m cells were used as control. Data are expressed as Absorbance values (OD 570 nm) +/- SD. (**B**) Murine RAW 264.7 macrophages were treated as in A in the presence or not of 50% neuroblastoma SKNAS conditioned medium (NBCM 50%). (**C**) LGALS3BP intracellular and secreted protein levels in knockdown (shLGALS3BP) vs control vector-infected (shCTR) SKNAS cells were analyzed using WB and ELISA, respectively. Note, secreted LGALS3BP was evaluated by ELISA in cells grown without puromycin before mice injection. D) Growth curve of subcutaneous hNB neuroblastoma xenograft model showing antitumor activity of intravenous 1959-sss/DM3 administration (arrows) at the dose of 10 mg/kg twice weekly for a total of 4 doses.

**
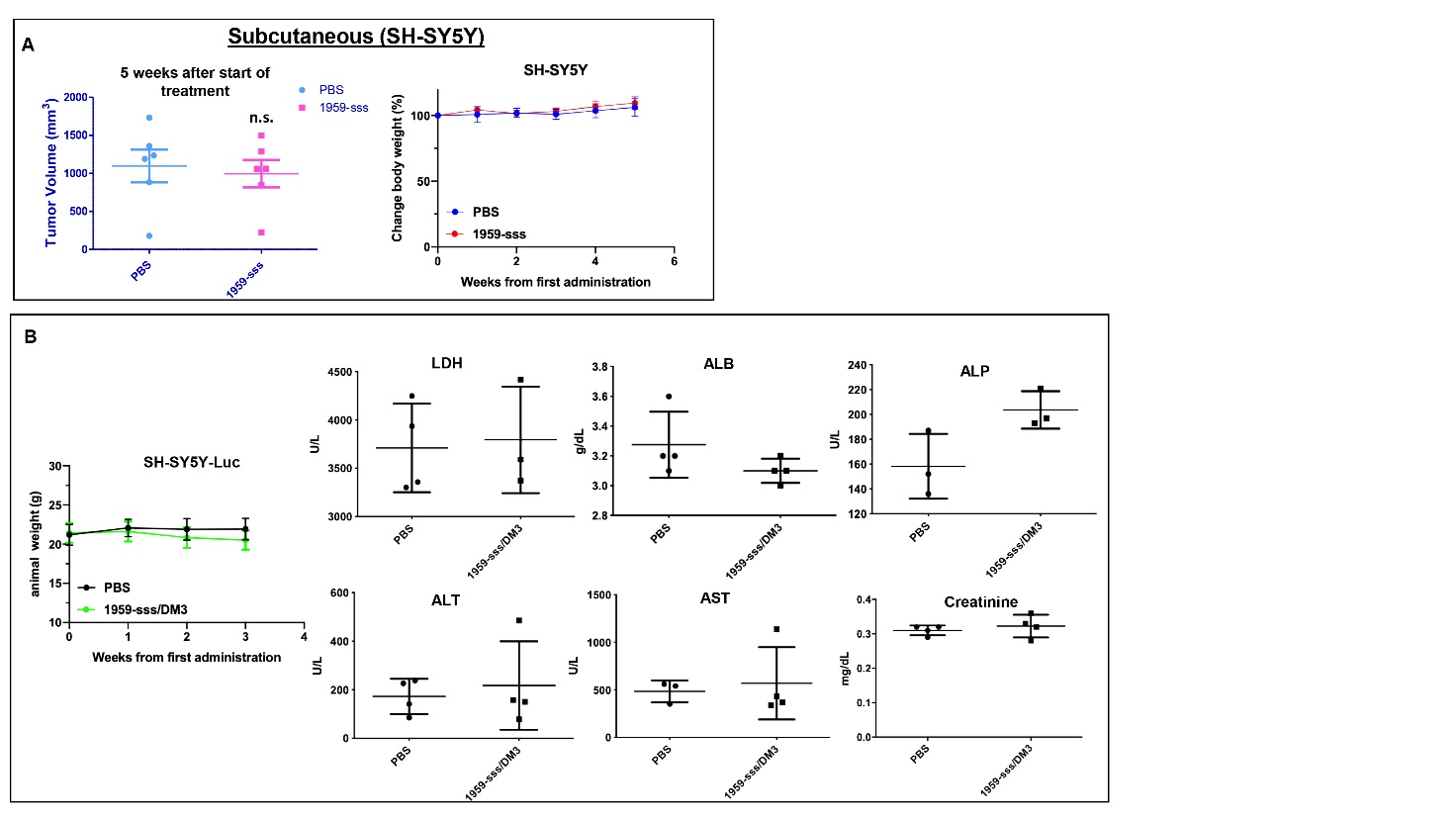
 Figure S3.** 1959-sss/DM3 is well tolerated in mice (**A**) Subcutaneous SH-SY5Y neuroblastoma xenograft model showing antitumor activity of intravenous 1959-sss administration at the dose of 10 mg/kg twice weekly for 4 doses. Graph shows tumor volumes 5 weeks after start of treatments (left panel) and percent change of body weight in mice during the five weeks after the start of treatment (right panel). (**B**) Body weight of mice during the three weeks after start of treatments (left panel); biochemical analysis of blood samples collected 24 h after the last treatment with 1959-sss/DM3 (right panel). LDH: Lactate Dehydrogenase; ALB: Albumin; ALP: Alkaline Phosphatase; ALT: Alanine Transaminase; AST: Aspartate Transaminase. Due to the low amount of available blood samples, some tests were performed for a limited number of mice.

| **A** | 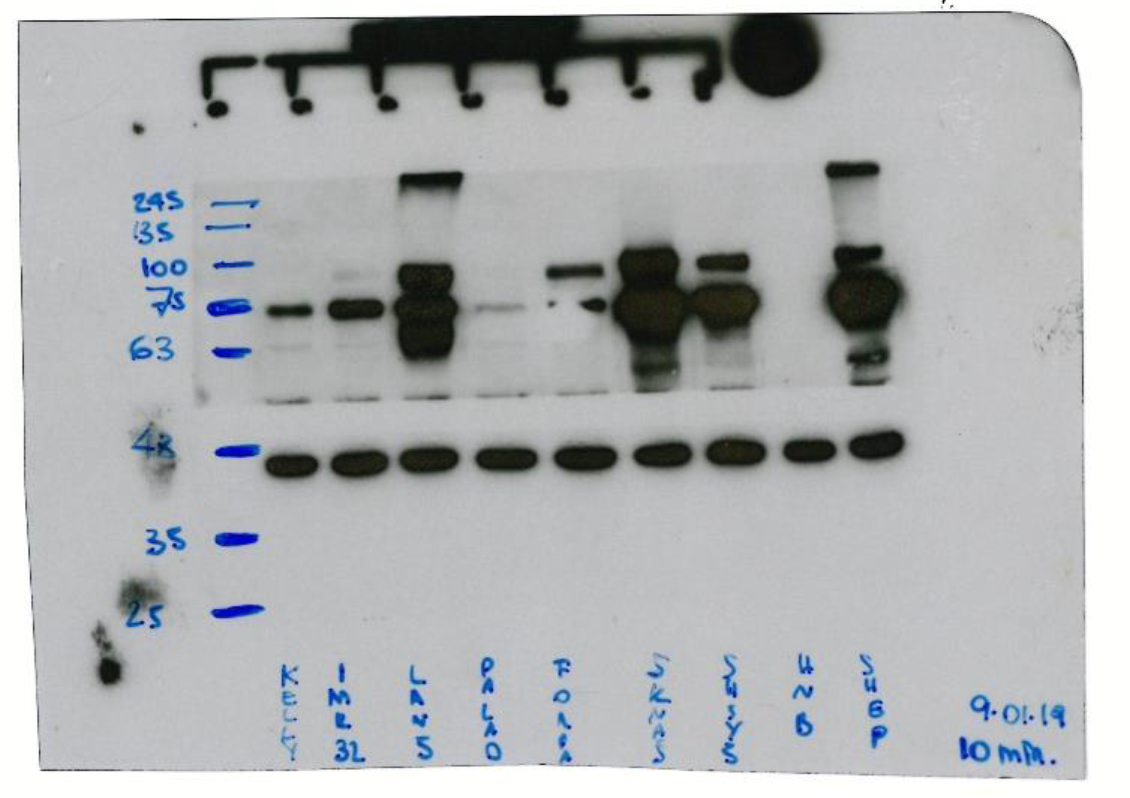 | **B** | 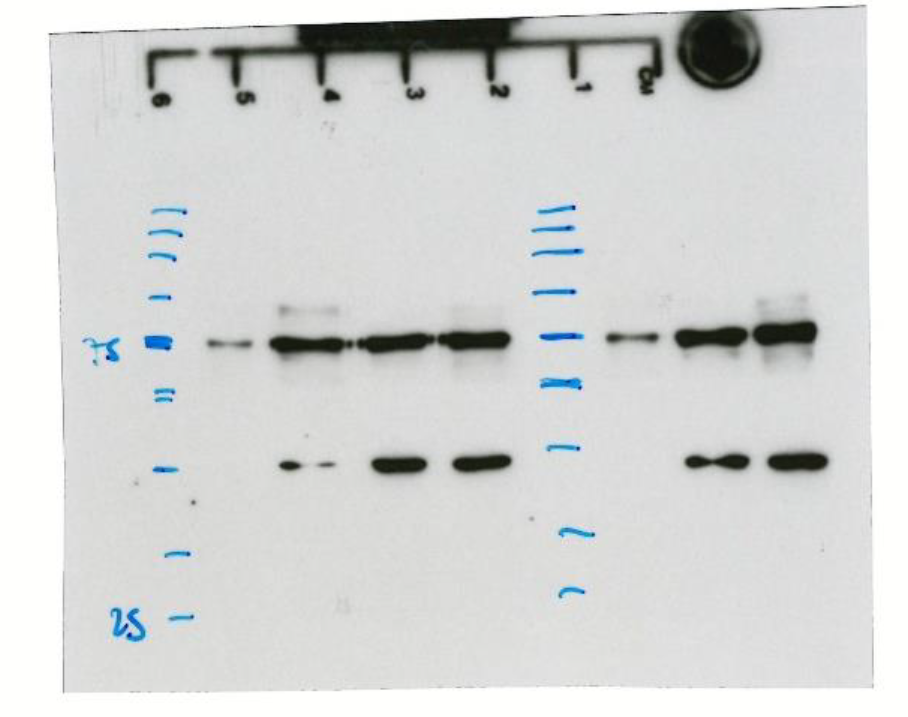 |
| --- | --- | --- | --- |
| **C** | 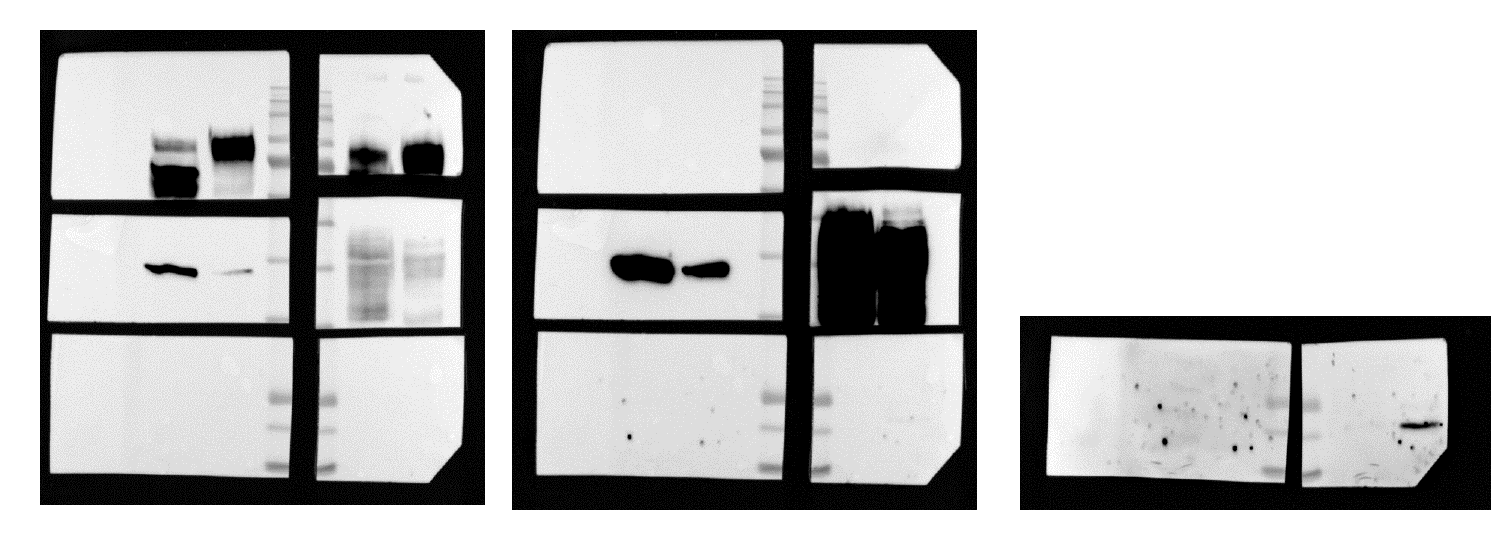 | | |
| **D** | 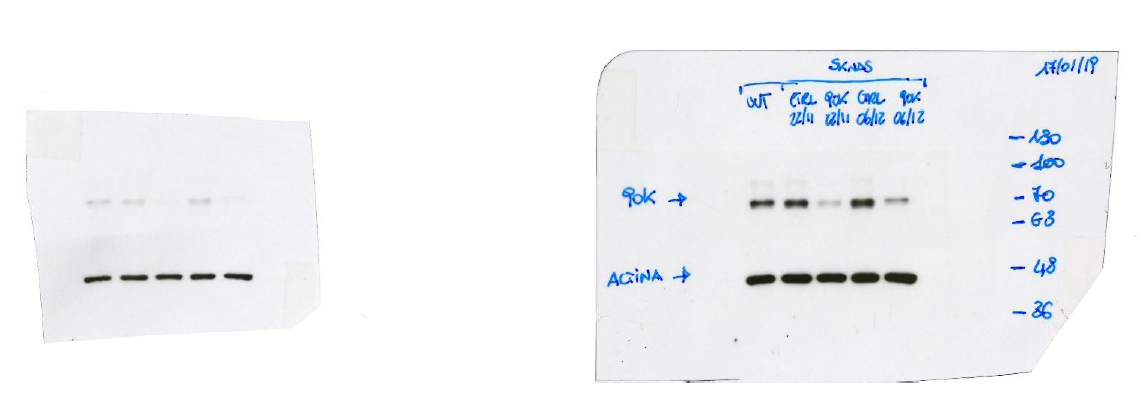 | | |

**Figure S4.** Uncropped Western Blotting figures: (**A**) The uncropped Western Blotting figure of Figure 1B (**B**) The uncropped Western Blotting figure of Figure 5B (**C**) Uncropped Western Blotting figures of Figure 1E (**D**) Uncropped Western Blotting figures of Figure S1B.
